# Multidrug-resistant tuberculosis (MDR-TB): an evolving threat to the Nigerian health system – a review

**DOI:** 10.1097/MS9.0000000000004312

**Published:** 2025-11-18

**Authors:** Aminu Shehu, Malik Olatunde Oduoye, Araj Naveed Siddiqui, Hareem Shaikh, Janice Odhiambo, Muhammad Muhsin, Abdullahi Zainab Zubairu, Chidiogo Ezenwoba, Ahmad Abdulhadi, Chrispin Biamba, Laiba Shakeel, Amidu Alhassan, Adolphe Karegeya, Mc Juan Muco Mugisha, Calvin R. Wei, Freddy Zihindula, Eric Buchangende Ndagano, Thierry Nasibu Ntumba, Christian Tague, Aymar Akilimali

**Affiliations:** a College of Natural and Pharmaceutical Sciences, Bayero University Kano, Kano State, Nigeria; b Department of Research, Medical Research Circle, Goma, DR Congo; c Department of Medicine, Jinnah Sindh Medical University, Karachi, Pakistan; d School of Pharmacy, University of Nairobi, Nairobi, Kenya; e College of Medical Sciences, Ahmadu Bello University, Zaria, Nigeria; f Faculty of Medicine, Afe Babalola University, Ado-Ekiti, Nigeria; g Department of Internal Medicine, Dow University of Health Sciences, Karachi, Pakistan; h College of Health and Allied Sciences, School of Nursing and Midwifery, University of Cape Coast, Cape Coast, Ghana; i College of Nursing, University of Utah, Utah, USA; j Faculty of Medicine, University of Rwanda, Kigali, Rwanda; k Clinical Research Department, Rinda Ubuzima Research Organization, Kigali, Rwanda; l Department of Research and Development, Shing Huei Group, Taipei, Taiwan; m Faculty of Medicine, Evangelic University in Africa, Bukavu, DR Congo; n Ecole de Santé Publique, Université Libre de Bruxelles, Bruxelles, Belgium; o Faculty of Medicine, Catholic University of Sapientia of Goma, Goma, DR Congo; p International Veterinary Vaccinology Network, The Roslin Institute University of Edinburgh, Edinburgh, United Kingdom

**Keywords:** antitubercular agents, humans, multidrug-resistant, Mycobacterium tuberculosis, Nigeria, tuberculosis, World Health Organization

## Abstract

Multidrug-resistant tuberculosis (MDR-TB) is an increasing public health issue that threatens the efforts in the treatment and control of tuberculosis (TB) worldwide, including in Nigeria. According to the World Health Organization, there were 558 000 MDR-TB cases globally in 2019. Nigeria, being the most populous country in Africa, is said to carry a larger proportion of MDR-TB in the world. If not addressed promptly, many Nigerian populations will continue to suffer from MDR-TB, which could result in increased morbidity and mortality rates. Eliminating the threat of MDR-TB in Nigeria requires a multifaceted approach that combines national and international efforts. These approaches should be centered on the molecular testing of MDR-TB using line probe assays and GeneXpert MTB/RIF technology, which enables early and efficient diagnosis of *Mycobacterium tuberculosis* and TB drug resistance among patients in high-risk populations. Improvements can occur through the development and implementation of new treatment therapies and investment in research to discover additional treatment options for TB. Public awareness and education about the disease are also important. If these recommendations are implemented, they can significantly decrease the burden of MDR-TB in Nigeria.

## Introduction

Multidrug-resistant tuberculosis (MDR-TB) is an increasing public health issue that threatens the efforts in the treatment and control of tuberculosis (TB) worldwide, including in Nigeria[1]. Globally, the prevalence of MDR-TB is predicted to be 3.3% among newly diagnosed TB patients and 17% among those who have already received treatment[2]. Nigeria is grappling with a substantial burden, with an estimated 4600 cases of MDR-TB reported in 2022, and an incidence rate of 4.3 per 100 000 population[1]. MDR-TB strains, which are resistant to first-line TB drugs, pose serious treatment challenges, often leading to poorer outcomes and higher mortality rates[3]. This situation is exacerbated by Nigeria’s high population density and the prevalence of HIV, which increases susceptibility to TB and complicates its management[4]. The growing prevalence of MDR-TB not only threatens the effectiveness of existing TB control efforts but also risks triggering a wider public health crisis if left unchecked. A strain of the bacteria that causes the disease is resistant to at least two of the most potent first-line TB treatments, isoniazid (INH) and rifampicin. This makes it more challenging and expensive to treat than the usual TB, and the course of treatment can last up to 2 years, involving numerous medications^[4,5]^. Close contact with individuals who already have the disease or latent infection with drug-resistant TB bacteria is the main mechanism for the spread of drug-resistant TB[6]. These persons, referred to as “carriers,” could unknowingly infect others if they go undiagnosed or untreated. The continued spread of drug-resistant TB worsens the global TB epidemic and imposes a tremendous burden on health systems[7]. Nigeria, being the most populous African country, is said to carry a larger proportion of MDR-TB compared to other countries[4]. If there are no interventions put into action, Nigeria could face ever-increasing rates of morbidity and mortality. This study complies with the TITAN guidelines[8].

### Aims and objectives

This review article presents current data on the prevalence of MDR-TB in Nigeria and discusses the role of Direct Observed Treatment Strategy (DOTS) and policies implemented by national and international agencies to reduce the burden of the disease. It further discusses the challenges encountered by Nigeria and the steps taken by the government to address these issues. This review article aims to

- Bridge knowledge gaps among the Nigerian population regarding the evolving threat of MDR-TB to the country’s health systems and suggest future directions to mitigate its spread.
- Support informed decision-making in Nigeria by providing critical insights.

## Methods

### Study design

We employed a narrative review methodology and synthesized existing literature on MDR-TB in Nigeria.

### Data sources

We performed a comprehensive literature review on PubMed, Scopus, ScienceDirect, and the World Health Organization Global Health Observatory with a focus on the current burden of MDR-TB in Nigeria, the challenges encountered, national and international strategies, and implications for future MDR-TB control.

### Keywords

Specific keywords, including *Multidrug-Resistant Tuberculosis, Rifampicin-resistant Tuberculosis, Nigeria, Mycobacterium tuberculosis, Direct Observed Treatment Strategy, and Mitigation Strategies*, were used with Boolean operators (AND, OR) to enhance the search strategy.

### Inclusion criteria

All types of peer-reviewed records, including original research, systematic reviews, meta-analyses, the World Health Organization (WHO) reports, and any other reports related to MDR-TB in Nigeria published between January 2010 and August 2025 were considered. Studies with only human subjects were included.

### Exclusion criteria

Non-peer-reviewed and animal studies were excluded. Studies in languages other than English and not focused on MDR-TB were excluded.

**HIGHLIGHTS**

- Multidrug-resistant tuberculosis (MDR-TB) is an increasing public health issue that threatens the efforts in the treatment and control of tuberculosis worldwide including in Nigeria.
- In Nigeria, MDR-TB primarily develops due to the inappropriate use of antituberculosis medications and the widespread misuse of antibiotics among the population.
- We believe that if all these recommendations are well implemented in Nigeria, Nigeria can record a zero-free MDR-TB burden.

### Data extraction

Key data were extracted from the selected studies and summarized in the article.

### Thematic framework

**Figure 1.**
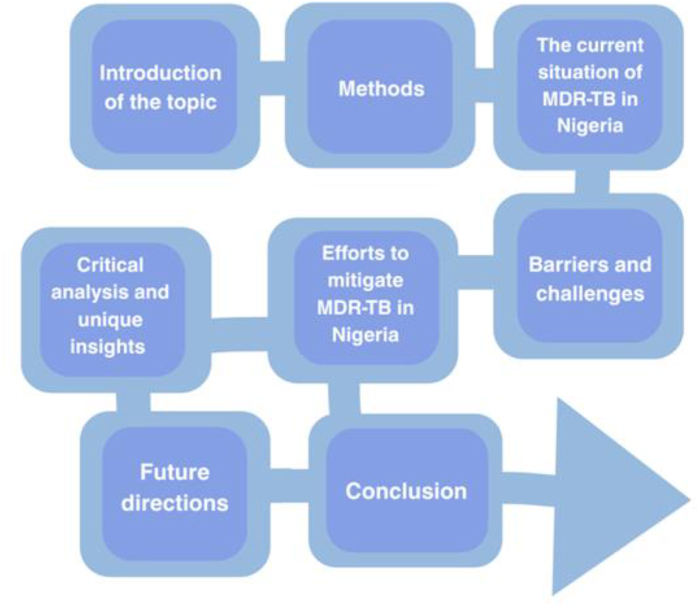
An outline of the concepts discussed in this article.

The thematic framework of the topics discussed in this article is shown in Figure 1.

## The current situation of MDR-TB in Nigeria

**Figure 2.**
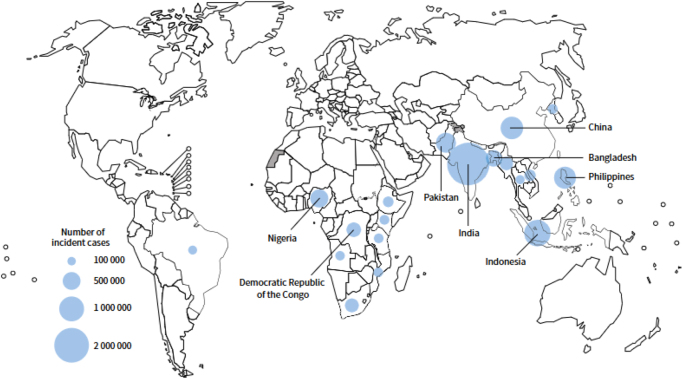
World map showing the estimated number of incident Tuberculosis cases in 2022 for countries with at least 100 000 incident cases. The larger the circle, the higher the number of incident TB cases. A legend in the bottom left corner gives a scale for interpreting the size of the circles: 100 000, 500 000, 1 000 000, and 2 000 000 incident cases.

In Nigeria, MDR-TB primarily develops due to the inappropriate use of antituberculosis medications and the widespread misuse of antibiotics among the population[1]. The emergence of MDR-TB in Nigeria has also been adversely affected by the transmission of HIV, which weakens the immune system[9]. Figure 2 shows the estimated number of incident TB cases in 2022 for countries with at least 100 000 incident cases.

According to estimates, 13% of global TB cases are caused by HIV coinfection[10]. A meta-analysis of patient data by Ahuja *et al* found that globally, treatment outcomes for MDR-TB were poor, with slightly more than half of patients achieving treatment success[11]. Treatment success was attributed to factors like: overall treatment duration, number of effective treatment options used, and the use of second-generation fluoroquinolones such as levofloxacin, lomefloxacin, moxifloxacin, gatifloxacin, and sparfloxacin. However, no association with hospitalization or ambulatory approach during the intensive phase was examined[11].

## Barriers and challenges driving the escalation of MDR-TB in Nigeria

**Figure 3.**
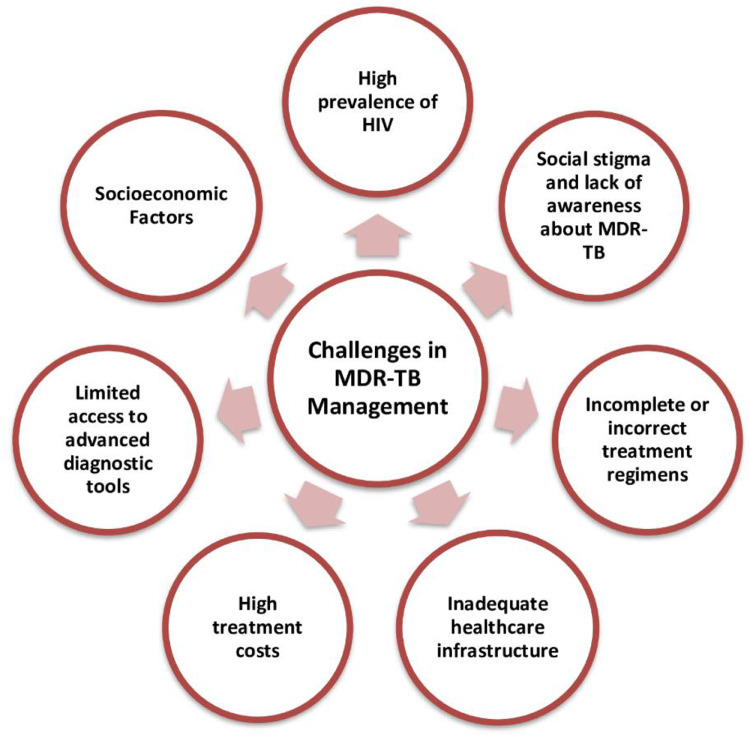
Major challenges in managing MDR-TB in Nigeria, including limited diagnostic access, high treatment costs, and inadequate healthcare infrastructure. Social stigma, high HIV prevalence, and socioeconomic factors further hinder effective treatment and control efforts.

The escalating MDR-TB situation in Nigeria is driven by several significant barriers and challenges. Limited access to advanced diagnostic tools and high treatment costs, compounded by inadequate healthcare infrastructure, restrict the timely and effective management of MDR-TB cases[1]. Incomplete or incorrect treatment regimens, along with the misuse of antibiotics and the prevalence of substandard drugs, contribute to the development and spread of drug-resistant strains[12]. Social stigma and lack of awareness about MDR-TB discourage timely diagnosis and adherence to treatment, while weak surveillance systems hinder accurate tracking and response^[13–15]^. Additionally, the high prevalence of HIV complicates treatment and exacerbates outcomes. Moreover, socioeconomic factors such as poverty, overcrowding, and rapid urbanization further facilitate the spread of MDR-TB^[15–17]^. These intertwined barriers and challenges create an out-of-control situation that demands a comprehensive and coordinated public health response. Barriers and challenges in the management of MDR-TB in Nigeria have been summarized in Figure 3.

## Efforts to mitigate MDR-TB in Nigeria

### The role of DOTS in mitigating MDR-TB

DOTS is given during the intensive phase (first 2 months) of TB to improve drug compliance and prevent multidrug TB resistance. DOTS therapy in Nigeria is started when there is a positive smear of TB or after the patient presents with signs and symptoms of TB^[18,19]^. Only first-line drugs (INH, Rifampicin, pyrazinamide, ethambutol) are used. Afterward, the patient is observed closely to ensure adherence to the first-line drugs that were prescribed at the health facility[20]. This is carried throughout the intensive phase of TB management and can be continued in the continuation phase. All patients diagnosed with TB receive DOTS daily in the TB Unit. Here, they are also educated about their disease and treatment course, and are observed for any complications. Home visits are conducted before the patient’s discharge, along with contact tracing and locating patients who have defaulted. Patients are followed up by physicians at 2 weeks, 1, 2, and 5 months, and as needed, unless they are “lost to follow-up” or transferred to another center[21]. In addition to DOTS, patients are encouraged to eat a balanced diet, sleep alone in well-ventilated rooms during the intensive treatment phase, and ensure that their sputa are appropriately disposed of to prevent the spread of TB to others[22]. DOTS therapy in Nigeria aims to prevent the development of drug-resistant organisms by improving drug compliance. Drug-resistant organisms are common in low- and middle-income countries like Nigeria due to co-existing HIV infection, overcrowding, inadequate nutrition, and low-quality health care[23]. Other aims of DOTS include slowing the progression of TB, preventing transmission of infection, achieving a cure, and preventing relapse. A limitation of this therapy is that culture and susceptibility are generally not determined in these health facilities before the therapy is started. Hence, drug resistance is not detected even if it is present. Since the introduction of DOTS therapy, the incidence of TB in some parts of Nigeria has drastically reduced. This is shown in a study conducted by UKwaja K *et al* at a hospital in Ebonyi State, Nigeria, which stated that DOTS significantly decreased the TB case notification rates in Ebonyi State. They further stated that there was a concomitant increase in the proportion of smear-negative cases compared to smear-positive pulmonary TB cases[22].

### National and international policy responses to MDR-TB in Nigeria

Although TB control and prevention efforts in Nigeria have progressed relatively well over the last two decades since the introduction of DOTS, this disease still poses a public health challenge to the Nigerian health system[24]. Experts in Nigerian TB control strategies highlighted two reasons why multidrug resistance continues to emerge and spread in Nigeria. These reasons include (1) mismanagement of TB treatment and (2) person-to-person transmission^[25,26]^. Over the years, the Centers for Disease Control’s on Antimicrobial Resistance (CDC-AR) and Solutions Initiative has tried as much as possible to invest in the national infrastructure to detect, respond, contain, and prevent resistant infections such as MDR-TB, histoplasmosis, aspergillosis, and other opportunistic fungal infections across most healthcare settings in Nigeria[27]. The National Action Plan for Combating Antibiotic-Resistant Bacteria between 2020 and 2025 proposed adopting the One Health approach in the fight against MDR-TB in Nigeria. This action aims to strengthen Nigerian healthcare services, particularly in the departments of public health, veterinary medicine, agriculture, food safety, and research and manufacturing[28]. Similarly, the WHO recommended expanded access to all-oral regimens in the treatment of TB worldwide in 2019[26]. The treatment success rate of diagnosing multidrug-resistant/rifampicin-resistant tuberculosis (MDR/RR-TB) patients was 60%. In the year 2020, the WHO also recommended a new shorter (9–11 months) and full oral regimen for patients with MDB-TB, in all Sub-Saharan African countries, including Nigeria[29]. Research findings have shown that patients find it easier to complete the shorter regimen compared with the longer regimens that last up to 20 months[30].

### Government-led initiatives and their impact on MDR-TB control in Nigeria

The Nigerian government has implemented a few initiatives to combat MDR-TB. Efforts include expanding the network of GeneXpert machines for rapid diagnosis, enhancing TB control programs through community-based care and active case finding, and launching public awareness campaigns to reduce stigma and promote treatment adherence^[31,32]^. Collaboration with international partners like the WHO and USAID has provided crucial funding and technical support, while integration of TB and HIV services aims to improve care for co-infected patients[32]. These initiatives have led to increased case detection and treatment adherence. However, challenges such as inconsistent implementation, resource constraints, and regional disparities continue to hinder overall effectiveness.

The Institute of Human Virology Nigeria has been active in TB control through interventions in health facilities that the Institute supports. These include scaling up TB prevention, diagnostic, and treatment services through private-sector engagement. The objective is to engage private and public healthcare providers in the fight against TB using international healthcare standards. The institute is working to reduce the burden of TB in the country and reach more than 60% of Nigerians who access health care in the private sector[33]. The TB epidemic has been under control with notable achievements over the years through the establishment of the Nigerian MDR-TB National Treatment Guidelines, the award of the Global Fund Round 9 grant (GFR9) for the containment of MDR-TB, and the crisis intervention of MDR-TB patients at medical facilities up to the Local Government Areas level are a few of these achievements. However, there are still many challenges, such as inadequate funding and a lack of trained healthcare workers. Sustainable Development Goals (SDG) Target 3.3 includes ending the TB epidemic by 2030. The End TB Strategy defines milestones (for 2020 and 2025) and targets (for 2030 and 2035) for reductions in TB cases and deaths[1]. Bold commitments to end TB by 2030 have been made by several countries in the Sustainable Development Goals, the WHO End TB Strategy, and the 2018 political declaration on the fight against TB. However, the epidemic shows no sign of slowing down in Nigeria^[34,35]^.

### Implications of the BCG vaccine and its facilitation in Nigeria

Bacille Calmette–Guerin (BCG) is currently the only licensed TB vaccine in the world. While it provides moderate efficacy in preventing severe forms of TB in infants and young children, it does not adequately protect adolescents and adults, who account for about 90% of TB transmissions globally[36]. Efforts to vaccinate newborn babies with BCG at birth in Nigeria have also been hampered. The reason is that most parents, especially mothers, still lack awareness of the importance of vaccination against TB. Other factors like insecurity, banditry, kidnapping, and Boko Harm insurgencies, especially in the Northeastern part of Nigeria, have sabotaged the efforts to fight against MDR-TB[37].

Dr Tedros Adhanom Ghebreyesus, the Director-General of the WHO, announced plans to establish a new TB Vaccine Accelerator Council, which will facilitate the licensing and use of effective novel TB vaccines, catalyzing high-level alignment between funders, global agencies, governments, and end users in identifying and overcoming barriers to TB vaccine development [22]. Unfortunately, this effort was not implemented in Nigeria. This is perhaps due to a lack of political will or poor logistics in the Ministry of Health[38].

## Critical analysis and unique insights from the Nigerian context

In a meta-analysis by Onyedum *et al*, MDR-TB accounted for 6% and 32% of new and previously treated cases, respectively[39]. This suggests that the frequency of MDR-TB in Nigeria is similar to the latest estimates from other high burden countries, including Iran, China, and Ethiopia. However, it is lower than the estimates reported from Burundi and Portugal^[40–44]^. The prevalence of 6% for new MDR-TB cases reported in this meta-analysis is higher than the estimates provided by the WHO (3.2–5.4%)[25]. This means there is a possibility of gross underestimation of cases from Nigeria. The burden appears to be the same in other parts of Africa. TB drug resistance was found to be 58.7% in Desta, Ethiopia[45]. Another study from Kinshasa, Congo, reported resistance to first-line drugs in 43.5% of the participants[46].

Most of the studies from Nigeria have focused on the prevalence of MDR-TB, facilitators and barriers in diagnosis and management, patients’ perspectives, ease and equity of access to care, timeliness of treatment initiation, and efficacy of short-term treatment courses^[39,47–51]^. However, Ogbuabor *et al* emphasized that fewer studies in Nigeria examine how health workers perceive the introduction and implementation of MDR-TB management at the subnational level and how fundamental elements of the Nigerian health system impact quality of care for these patients[52]. Studies on healthcare infrastructure, healthcare worker training, and their perspectives are urgently needed. We also need to understand patients’ treatment choices, their knowledge of healthcare rights, and the effectiveness of support systems to improve treatment adherence.

Mamuda *et al* examined the molecular characterization of MDR-TB in Kaduna State, Nigeria, and found that multiple mutations in the *rpoB* gene are responsible for the high prevalence of MDR-TB. There is a need for extensive molecular-based surveillance from Nigeria to fully understand the role of specific mutations in genes, including *rpoB, katG*, and *inhA* in conferring resistance to rifampicin and INH[53]. This will help us comprehend the extent of MDR-TB transmission. Furthermore, Onyedum *et al* suggested that a large-scale nationwide survey on antituberculosis drug resistance should be conducted to strengthen disease detection and programmatic management[39].

## Future directions

In response to the threat of MDR-TB to the effective control and prevention of TB in Nigeria, The Federal Ministry of Health must strengthen its collaborations with the WHO and the CDC, as well as other TB control organizations in the world, in putting well-implemented action plans and health structures for the initiation and expansion of the programmatic management of drug-resistant TB and policies in Nigeria through a functional national MDR-TB committee in the country[24]. The Nigerian policymakers should create renewed energy in expanding the BCG vaccination coverage and acceptability among Nigerian parents, especially in rural areas. This would be achievable through mass campaigns and education by Nigerian healthcare providers. These healthcare providers should ensure engagement with local stakeholders and give credibility to antimicrobial resistance communication campaigns. Participants should be encouraged to underline the importance of involving local institutions, both official and customary systems, in Nigeria. In many rural areas, the authority of traditional or tribal leaders, including religious leaders, is strong, which is why these leaders in Nigeria should be involved in meetings with local farmers via local agricultural advisors or state veterinary offices[54].

The Nigerian government should join other world-leading countries in the quest for more effective TB vaccines other than BCG by leveraging the WHO’s convening power and experience in fostering partnerships[38]. All the approved health laboratories in Nigeria should improve their capacities for early detection and prompt diagnosis of MDR/RR-TB in suspected and confirmed patients with TB with sophisticated GeneXpert machines[54]. Early detection and prompt diagnosis of TB have been proven to yield effective treatment^[1,9,24,31]^.

Nigeria, being the first country in West Africa to provide people with TB access to the groundbreaking new antituberculosis regimen like Bedaquiline, Pretomanid, and Linezolid treatment (BPaL) under operational research conditions, is a plus for the fight against MDR-TB in the country[33]. The Nigerian federal government needs to willingly distribute these new regimens to all the healthcare centers in the country, especially the primary health care centers. Resistance to fluoroquinolones should be excluded before the initiation of treatment with this regimen. The BPaL regimen is a 6-month, three-drug, oral treatment therapy meant for people with advanced forms of drug-resistant TB and is expected to have a higher success rate than previous treatments with four to six medicines that lasted at least 18 months. The BPaL regimen was developed by the TB Alliance and was implemented in Nigeria by the National TB Program, with support from the KNVC Tuberculosis Foundation. The BPaL regimen has likewise been approved by the U.S Food and Drug Administration, the European Medicines Agency, the Drug Controller General of India, and recommended by the WHO under operational research conditions. However, the new BPaL regimen (6 months, all oral) has shown a cure rate of 90% in this patient group, according to Phase 3 trial results published in the *New England Journal of Medicine*[33].

Eliminating the threat of MDR-TB in Nigeria requires a multifaceted approach (public health, global health, and One Health strategy) that combines efforts. All these approaches should be centered on the molecular testing of MDR-TB using line probe assays and GeneXpert MTB/RIF technology, which enables early and efficient diagnosis of *Mycobacterium tuberculosis* and TB drug resistance among patients in high-risk populations in Nigeria. Improvements can happen with the development and usage of new treatment therapies and investment in research to find more treatment options for TB. Public awareness and education about the disease are also important[22].

## Conclusion

The MDR-TB burden in Nigeria remains a major public health concern, but the country is positioned at a critical juncture where recent advancements can be leveraged for sustained progress. With the adoption of molecular diagnostic tools, the introduction of new regimens such as BPaL, and the support of global partners, Nigeria has demonstrated its capacity to implement innovative strategies for TB control. However, the persistence of systemic barriers, including inadequate funding, gaps in laboratory infrastructure, shortages of skilled health workers, and weak integration of private-sector care, continues to undermine national targets. Progress toward elimination will require a multisectoral strategy that combines public health, global health, and One Health approaches, while ensuring that implementation is phased, realistic, and tailored to Nigeria’s context. Importantly, the lessons from Nigeria have wider regional and global significance, as they highlight how high-burden countries can integrate advanced therapies, strengthen diagnostic networks, and engage communities in culturally sensitive ways. By addressing these challenges in a coordinated and evidence-driven manner, Nigeria can achieve measurable and sustained reductions in MDR-TB incidence and mortality, align with the End TB Strategy and Sustainable Development Goal 3.3, and contribute to the global push toward ending the TB epidemic by 2030. While immediate elimination may not be feasible, a decade-long phased roadmap that prioritizes diagnostics, equitable treatment distribution, and vaccine innovation represents a hopeful path forward.

## Limitations of the study

Our article is limited by data availability and reliability issues due to incomplete or inconsistent reporting across different regions. Diagnostic challenges, such as limited access to advanced tools and delayed diagnosis, hinder accurate case identification. The healthcare system’s constraints, including resource limitations and poor patient follow-up, further complicate effective disease management. Socioeconomic and cultural factors, like stigma and traditional beliefs, can lead to underreporting and treatment noncompliance. Additionally, regional variations in MDR-TB epidemiology, the impact of HIV coinfection, and potential selection bias limit the generalizability of findings.

## Data Availability

Not applicable.
